# Solitary Fibrous Tumor of Head and Neck Region: A Series of Three Cases at an Uncommon Location With a Review of the Literature

**DOI:** 10.7759/cureus.58213

**Published:** 2024-04-13

**Authors:** Muhammad Usman Tariq, Abdullah Alsulaiman, Ammara Kashif, Eman Keshk, Salwa H Alhassani, Hessa Alkhudaidi

**Affiliations:** 1 Histopathology Unit, Laboratory Department, Al Hada Armed Forces Hospital, Taif, SAU; 2 Laboratory Department, Al Hada Armed Forces Hospital, Taif, SAU; 3 Genetics Division, Laboratory Department, Al Hada Armed Forces Hospital, Taif, SAU; 4 Forensic Toxicology Section, Laboratory Department, Al Hada Armed Forces Hospital, Taif, SAU

**Keywords:** recurrence, cd34, storiform pattern, head and neck, solitary fibrous tumor

## Abstract

Solitary fibrous tumors (SFTs) uncommonly involve the head and neck region. Head and neck SFTs (HNSFTs) exhibit diverse histological features and can mimic several neoplasms with different treatment and behavior. Herein, we report the clinicopathological features of three cases of HNSFT. Case 1 was a 29-year-old female who presented with a nasal cavity mass measuring 3.5 cm. The patient underwent surgical excision. Microscopic examination revealed classic histological and immunohistochemical (IHC) features of SFT. Unusual histological features included epithelioid morphology, clear cells, and edematous change. She developed local recurrence after 11 months, which was also treated with surgery. Case 2 was a 55-year-old male who developed a 1-cm mass at the buccal mucosa. Surgical excision of the tumor was performed. The tumor was completely circumscribed microscopically. Characteristic histological and IHC features of SFT were identified. Unusual histological features observed were an adenomatous pattern, clear cells, and myxoid change. The patient was alive and disease-free at the 12-month follow-up. Case 3 was a 59-year-old female presenting with a medial canthus mass measuring 1.4 cm. The patient underwent surgical excision. Histological and IHC features observed were diagnostic for SFT. Unusual histological features identified were wavy nuclei and multinucleated stromal giant cells. The patient was alive and disease-free at the 124-month follow-up. Diagnosis of SFT can be challenging in unusual locations like the head and neck region. In addition, the histological spectrum of HNSFT is diverse. Therefore, knowledge about unusual histological features and classic IHC expression is essential for establishing correct diagnosis. Long-term follow-up is recommended because of the risk of recurrence in HNSFT.

## Introduction

Solitary fibrous tumors (SFTs) are fibroblastic neoplasms that demonstrate intermediate (rarely metastasizing) behavior. Initially, SFTs were considered to occur specifically in the lung and pleura, but it is now known to occur at almost every anatomic site. Some of the more frequently involved extrapleural sites include the meninges, abdominal cavity, extremities, and head and neck region (HNR) [1]. Head and neck SFTs (HNSFTs) comprise 6-18% of all SFTs and approximately a quarter of extrathoracic SFTs. Within the HNR, more frequently involved sites include the sinonasal tract, orbit, oral cavity, and neck [2-5]. Given the diversity of its morphological features, SFT is called “the great simulator” as it can mimic several benign and malignant soft tissue tumors, creating diagnostic challenges at unusual locations like the HNR [2,6]. CD34 and STAT6 are useful immunohistochemical (IHC) markers for establishing the diagnosis of SFT [2,4,5,7,8]. In HNSFTs, recurrence and distant metastasis rates can be as high as 40% and 16.7%, respectively [2,9]. Because of its rarity, knowledge about the clinical behavior and treatment of HNSFTs is limited [2,4].

The aim of our study was to describe the demographic, histological, and immunohistochemical (IHC) features of three HNSFT cases and discuss the differential diagnoses in the HNR region.

## Case presentation

Case 1

**Figure 1:**
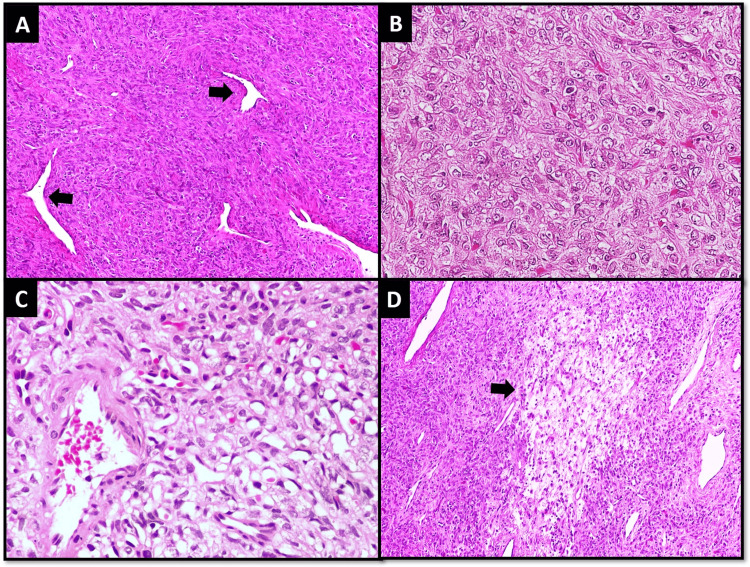
Case 1; Histological features (A) Tumor cells arranged in vague storiform and short fascicular pattern along with intervening staghorn (HPC-like) vessels (arrows). (B) Tumor cells showing epithelioid morphology. (C) Clear cell change. (D) Edematous change in stroma (arrow).

A 29-year-old female presented with nasal obstruction for two months. A CT scan revealed a vaguely circumscribed tumor measuring 3.5 x 2 x 1.4 cm involving the left side of the nasal cavity. The patient underwent surgical excision of the tumor. Microscopic examination revealed a partially circumscribed tumor composed of sheets and fascicles of spindle cells against collagenous background stroma containing hemangiopericytoma-like (HPC-like) vasculature. The tumor cells focally showed epithelioid morphology and clear cell change. The mitotic count was 1/10 high power field (HPF). Necrosis was not identified. Focal edema of the background stroma was also noted (Figure 1).

On IHC, the tumor cells were positive for STAT6, CD34, CD99 and BCL2. Cytokeratin AE1/AE3, smooth muscle actin (SMA), desmin, and S100 were negative. Hence, the diagnosis of SFT was established. The surgical margin was clear. The patient was categorized as low risk, according to the WHO classification of Soft Tissue and Bone Tumors, 5th edition [1]. The patient did not receive any adjuvant treatment. She developed local recurrence after 11 months, for which she underwent re-excision. The morphology of primary and recurrent tumors was similar.

Case 2

**Figure 2:**
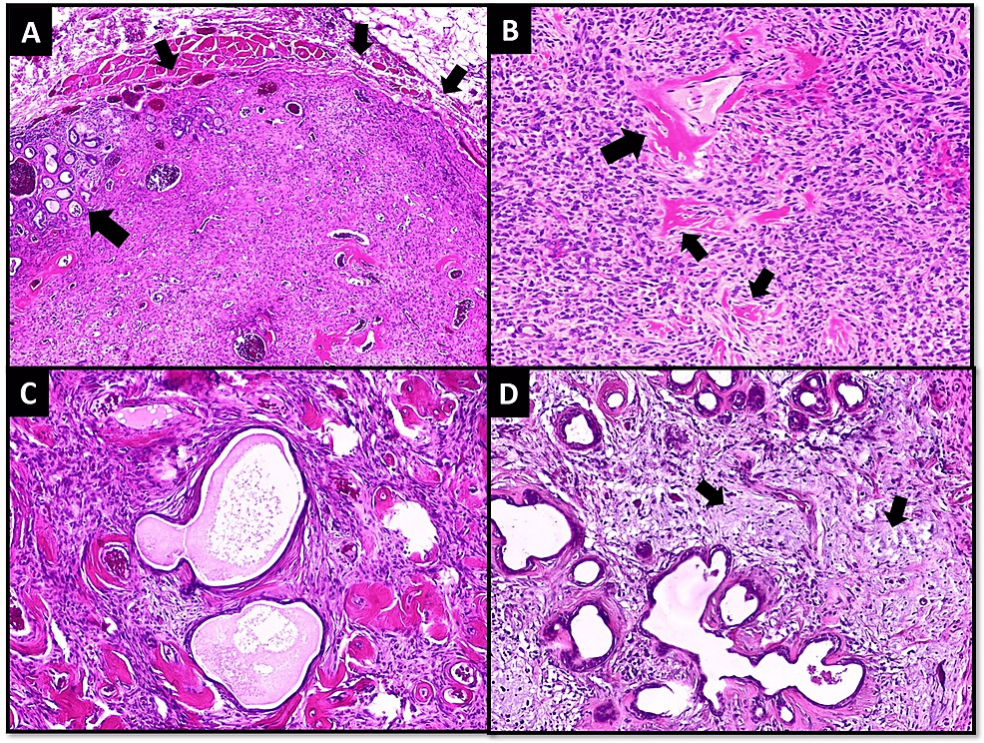
Case 2; Histological features (A) Low power view of the tumor showing well-circumscribed borders (smaller arrows) and entrapped minor salivary glands (larger arrow). (B) Collagen deposition in perivascular (larger arrow) and stromal (smaller arrows) locations. (C) Adenofibromatous pattern; tumor cells are encircling entrapped native salivary gland ducts. (D) Myxoid change in stroma (arrows).

A 55-year-old male presented with painless swelling at the left buccal mucosa for seven months. Imaging revealed a well-circumscribed 1 x 1 cm tumor involving the left cheek. The patient underwent surgical excision of the tumor. Microscopic examination revealed a completely circumscribed tumor composed of haphazardly arranged short fascicles of spindle cells against collagenous background stroma. Perivascular and stromal collagen deposition was appreciated. Focally, tumor cells were arranged around entrapped dilated minor salivary gland ducts to impart an adenofibromatous pattern. Focal clear cell change and myxoid stroma were also identified. The mitotic count was < 1/10 HPF. Necrosis was not identified (Figure 2).

On IHC, tumor cells were positive for STAT6 and CD34 but negative for SMA, S100, and cytokeratin AE1/AE3. The findings were consistent with SFT. The surgical margin was clear. The patient was categorized as low-risk and did not receive any adjuvant treatment. He was alive and disease-free at the 12-month follow-up.

Case 3

**Figure 3:**
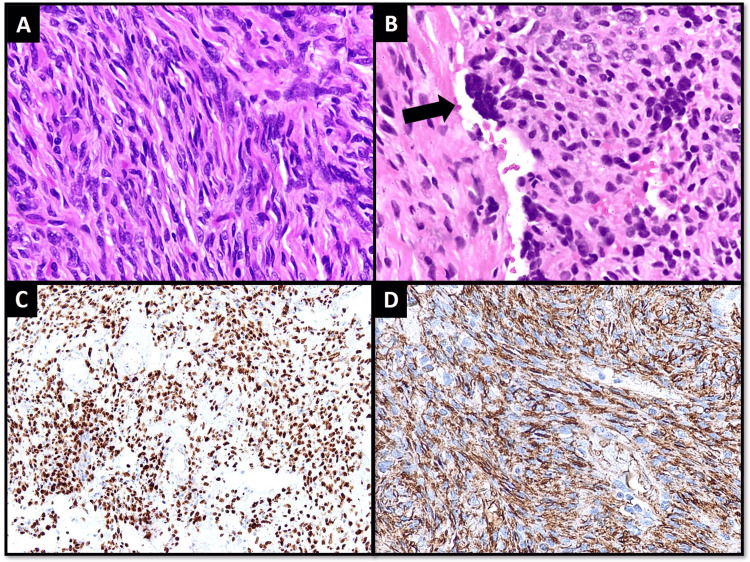
Case 3; Histological features and IHC stains (A) Tumor cells are showing wavy nuclei. (B) Multinucleated stromal giant cell (arrow). (C) Positive expression for STAT6 and (D) CD34 IHC stains.

A 59-year-old female presented with a slowly growing painless swelling at the medial canthus for one year. Imaging revealed a vaguely circumscribed soft tissue density mass in subcutaneous tissue measuring 1.4 x 1 cm. The patient underwent surgical excision of the tumor. Microscopic examination revealed a partially circumscribed tumor composed of short fascicles of spindle cells against background stroma showing HPC-like vasculature. Tumor cells focally showed wavy nuclei. Multinucleated stromal giant cells lining pseudovascular spaces were also identified. The mitotic count was 2/10 HPF. Necrosis was not identified. The tumor cells demonstrated positive expression for STAT6 and CD34 IHC stains (Figure 3).

Negative expression was observed for SMA, desmin, and S100 IHC stains. Hence, the diagnosis of SFT was made. The surgical margin was not clear. The patient was characterized as low-risk and did not receive any adjuvant treatment. She was alive and disease-free at the 124-month follow-up.

## Discussion

HNSFTs can occur in patients ranging from eight months to 94 years old without any gender predilection [2-4,7,8]. The majority of patients are middle-aged individuals, and the mean ages at presentation reported in the literature are 43.9-54 years [2-5]. All patients in our study were adults (median age: 55 years). We observed female predominance in our cohort. In larger series of HNSFT, the most common tumor sites were the sinonasal tract, neck, oral cavity, and orbit [2-5,7].

Tumors of the oral cavity are typically well-circumscribed [10]. In the sinonasal tract (SNT), tumors can either involve the nasal cavity or paranasal sinuses alone or in combination. Ethmoid and sphenoid sinuses are commonly involved [8]. In the orbital and periorbital location, tumors can involve intraconal and extraconal spaces of the orbit along with the sclera, conjunctiva, lacrimal sac, and eyelids [11]. Tumors in our series also occurred at common locations of HNSFT. The oral cavity tumor in our study was also completely circumscribed.

Most patients present with a swelling and symptoms related to the mass effect of having a tumor in a certain location, such as nasal obstruction, epistaxis, proptosis, ptosis, change in voice, upper respiratory distress, visual field changes, and headache [1,4,8,12,13]. Only 11% of patients experience pain, and pain is associated with larger tumor size [4]. The patients of our cohort also presented with a painless mass.

Radiologically, these tumors are well-defined, but local infiltration into adjacent bones, nerves, and blood vessels is seen in 13% of cases [4]. CT scans with contrast reveal variable degrees of heterogenous enhancement. MRI scans show T1W isointense and T2W hypointense signals [14].

Macroscopically, SFTs are usually well-circumscribed, partially encapsulated, solid, and fibrous with tan, white, grey, or reddish-brown cut surfaces [2,3]. Tumor sizes range from 0.4-18 cm. The median tumor size reported in different studies is 2.5-4 cm [2,4,5,11]. The median size of tumors in the oral cavity (2 cm) is significantly smaller than the median size of tumors in the sinonasal tract and neck (5 cm) [4]. Generally, small HNSFTs get noticed due to easy visibility and proximity to adjacent structures [4]. Tumor size was < 5 cm in all cases of our series.

Initially, SFT and hemangiopericytoma were described as separate entities. Later on, due to clinicopathological and histomorphological overlap, SFT and hemangiopericytoma were considered two ends of a histological spectrum. Identification of NAB2-STAT6 gene unified these entities which was followed by the adoption of SFT terminology for all of these tumors and the abandoning of hemangiopericytoma terminology [15]. Microscopically, tumors show patternless arrangements of ovoid to spindle-shaped cells with intervening staghorn-shaped (HPC-like) blood vessels and collagen bundles. Cellular tumors may show storiform, fascicular, or sheet-like patterns. Tumor cells usually have small, round to oval nuclei with vesicular chromatin and inconspicuous nucleoli [3,5,7,8,15]. Tumor cells may show cytologic atypia in up to 23% of cases and epithelioid morphology in 15% of cases [7]. Mitotic count ≥ 4/10 HPF has been reported in 18% of cases [10]. Background stroma may show cracking artifact and abundant keloid-like collagen [3,7,8,16]. Medium-sized blood vessels with surrounding fibrosis or hyalinization are commonly observed in SFT [5,7,8,15].

Some of the less frequent histological features that contribute to the histological spectrum of SFT include infiltrative growth (26-49% of cases), micro- and macrocystic spaces (7-43%), myxoid change (3-45%), lipomatous change (3-19%), giant cell angiofibroma-like areas (17%), stromal giant cells (7-10%), coagulative tumor necrosis (6%), nodular hyaline deposits (resembling collagen rosettes or amianthoid fibers; 5%), and dedifferentiation (1%) [2,3,5,7,16]. We also observed the classic morphological features of SFT in all cases as well as diversity of histological spectrum in at least some cases of our cohort.

The molecular hallmark of SFT is recurrent NAB2-STAT6 gene fusion, and STAT6 IHC expression is observed in almost all cases [2,4,5,7,8,17]. STAT6 IHC stain is particularly helpful in diagnosing CD34-negative SFT; however, STAT6 expression can be observed in other mesenchymal tumors, but SFT typically expresses nuclear positivity [4,7,15]. CD34 expression is observed in 90-98% of cases [2,4,5,7]. In HNSFT cases with loss of CD34 expression, malignant mesenchymal lesions come into the differential diagnoses such as biphenotypic sinonasal sarcoma (BSS), sinonasal sarcoma (SS), and malignant peripheral nerve sheath tumor (MPNST) [7]. A combination of STAT6 and CD34 should always be used and interpretations be made in light of morphological features and the results of other IHC stains [7]. Focal/multifocal expression for CD99, BCL2, SMA, S100, EMA, and pan cytokeratin is also seen in 75-94%, 91-93%, 6-16%, 8%, 2-5%, and 4% of cases, respectively [2,4]. HNSFTs are negative for desmin, NKX2.2, SOX10, CD31, ERG, synaptophysin, and chromogranin A IHC stains [3]. Some of the dedifferentiated SFT may also lack STAT6 IHC expression due to lack of chimeric protein. The possibility of SFT should be ruled out in such cases as molecular testing to NAB2-STAT6 gene fusion reveals positive results and confirms the diagnosis [18].

The histological spectrum of HNSFT broadens due to variations in cellularity and stromal changes [2]. Lipomatous SFTs can resemble spindle cell lipoma (SCL), which is composed of a mixture of bland spindle cells against myxoid stroma and mature adipocytes along with ropy collagen bundles. Tumor cells demonstrate positivity for CD34 and SMA, but STAT6 is negative. SCL is also characterized by loss of retinoblastoma (RB) gene expression [15]. Sinonasal glomangiopericytoma (SG) is a close differential diagnosis of SFT in the sinonasal tract. SG is composed of bland, uniform spindle cells with intervening staghorn vasculature. It lacks hypo- and hypercellular areas, thick collagen bundles, and perivascular hyalinization. Tumor cells demonstrate diffuse positive expression for SMA, muscle-specific actin, and β-catenin (nuclear expression) and negative expression for STAT6 and CD34. Nuclear β-catenin expression may also be seen in HNSFT [8]. BSS is a low-grade sarcoma composed of bland spindle cells arranged in intersecting fascicles with a herring-bone pattern, intervening thin collagen, and staghorn vasculature. Tumor cells are characteristically positive for SMA and S100 IHC stains. Invagination of the hyperplastic respiratory surface epithelium is a helpful distinguishing feature. Focal weak expression for CD34, desmin, MyoD1, and myogenin may be observed, while SOX10 expression is negative in these tumor cells [3,8]. Tumor cells may show cytoplasmic expression for STAT6, but nuclear expression is not observed [7]. The characteristic molecular alteration in BSNS is PAX3-MAML3 gene fusion [3]. Nasopharyngeal angiofibroma can mimic SFT given its rich vasculature surrounded by bland stromal cell component. The stromal cells are positive for SMA, androgen receptors, and β-catenin and negative for STAT6 IHC stains [3,7]. Poorly differentiated and monophasic forms of SS can mimic SFT because SS is composed of spindle to ovoid cells arranged in fascicular and sheet-like growth patterns with intervening staghorn vasculature and wiry collagen bundles. Positive CD99 and BCL2 IHC expression is shared by SS and SFT. Rare SS cases with STAT6 expression are also reported, but CD34 is always negative. Some cases of SFT may express weak positivity for TLE1, but diffuse positive expression favors SS. Translocation t(X;18) is the molecular signature of SS and is useful in diagnosing challenging cases [8,15]. MPNST may mimic SFT because of alternating hypo- and hypercellular areas, focal HPC-like patterns, and CD34 positivity, especially in low-grade cases. Focal nerve sheath morphology is seen in most cases. Loss of H3K27me IHC expression; STAT6 positivity; and positive expression of S100, SOX10, and GFAP favors MPNST [15]. Benign peripheral nerve sheet tumors such as schwannoma and neurofibroma can also be confused with SFT. Like SFT, schwannoma also shows alternating hypo- and hypercellular areas and hyalinized blood vessels; however, wavy nuclei with tapered ends can be a useful diagnostic clue. Neurofibroma can also pose challenges due to CD34 positivity. Schwannoma and neurofibroma are diffusely positive for S100 and SOX10 and negative for STAT6 [8].

Anaplastic carcinoma is the closest differential diagnosis in malignant SFT cases involving the neck and thyroid gland [2]. Aberrant PAX8 expression in SFT further complicates the condition and may lead to misdiagnosis. Diffuse pan-cytokeratin expression and lack of STAT-6 expression favors anaplastic carcinoma over SFT [2]. In the submucosal location, spindle cell carcinoma should always be ruled out. Malignant melanoma can also involve submucosal locations in the HNR and therefore should be kept in differentials [8]. Cellular SFT may resemble small, round-cell tumors [3].

The traditional concept of benign and malignant SFT has evolved over the years due to a lack of correlation with clinical behavior, and several risk-assessment models have been proposed [1]. The most widely practiced model by Demicco et al. predicts the risk of metastasis and tumor-related death and divides tumors into low, intermediate, and high-risk groups based on the patient’s age, tumor size, mitoses/mm2, and necrosis [7,19]. According to Smith et al., Demicco et al.’s model is of limited utility in the HNR because of the higher recurrence rate (~40%) and lower metastatic rate (~6%) in contrast to the higher metastatic rate (26%) and lower recurrent rate (10%) in locations outside the HNR [2]. They proposed an additive joint prognostic model conferring tumor size (> 5 cm) and mitotic count (> 4/10 HPF) to be associated with the risk of recurrence [4]. Thompson et al. also proposed a fairly accurate risk stratification model specifically for orbital tumors based on age (> 45 years), tumor size (> 3 cm), > 4 mitoses/2mm2, necrosis, moderate to high cellularity, and moderate to severe pleomorphism [20]. In one of the studies, positive surgical margin was the only factor predictive of time to recurrence [4]. In contrast, another study reported positive surgical margins in up to 67% of cases, but it was not associated with local recurrence [4]. Obtaining negative surgical margins is a challenge in tumors of the HNR, and margins should be interpreted with surgical and pathological correlation [2].

The recurrence rate in different studies has ranged from 0-40% [2,3,5,10,16,17,20]. Median time to recurrence ranges from 12-36 months [2,3,5]. Recurrence is less frequently observed in tumors involving the oral cavity and floor of the mouth [10,16]. Distant metastasis has been reported in 2.1-16.7% of cases, and it may develop 4-5 months after initial tumor resection [2,4,7,9,20]. Death from disease can occur in up to 8% of cases [2]. All our patients were categorized as low-risk. Surgical margins were positive in two cases. None of the patients received any adjuvant treatment. We observed recurrence in a single case, although none of the patients developed distant metastasis or died of disease. The primary and recurrent tumors did not exhibit any aggressive clinicopathological or histological features except for a positive surgical margin.

Surgical excision is the main treatment modality. Adjuvant therapies include postoperative radiation therapy and preoperative tumor embolization therapy [4]. Postoperative radiation therapy is administered more frequently to patients with recurrence, aggressive histological features, positive surgical margins, and local invasion [3,4].

## Conclusions

SFT can involve different sites in the HNR, such as the nasal cavity, buccal mucosa, and face. Microscopically, these tumors show at least some degree of circumscription. Apart from characteristic histological features, tumors show a variety of unusual histological features, such as epithelioid morphology, clear cells, wavy nuclei, multinucleated stromal giant cells, adenomatous patterns, and myxoid and edematous changes. These features can be misleading, and knowledge about these features helps avoid misdiagnosis. STAT6 and CD34 are the most useful IHC makers for establishing the diagnosis. HNSFTs could recur locally; therefore, close clinical follow-up should be maintained for a long duration.

## References

[1] Demicco EG, Fritchie KJ, Han A (2019). Solitary fibrous tumour. World Health Organisation Classification of Soft Tissue and Bone Tumours, 5th edition.

[2] Smith SC, Gooding WE, Elkins M (2017). Solitary fibrous tumors of the head and neck: a multi-institutional clinicopathologic study. Am J Surg Pathol.

[3] Baněčková M, Martínek P, Skálová A, Mezencev R, Hadravský L, Michal M, Švajdler M (2020). Solitary fibrous tumors of the head and neck region revisited: a single-institution study of 20 cases and review of the literature. Hum Pathol.

[4] Stanisce L, Ahmad N, Levin K, Deckard N, Enriquez M, Brody J, Koshkareva Y (2020). Solitary fibrous tumors in the head and neck: comprehensive review and analysis. Head Neck Pathol.

[5] Tariq MU, Asghari T, Armstrong SM, Ahmed A, Fritchie K, Din NU (2023). Solitary fibrous tumor of head and neck region; A clinicopathological study of 67 cases emphasizing the diversity of histological features and utility of various risk stratification models. Pathol Res Pract.

[6] Machado I, Nieto-Morales G, Cruz J (2020). Controversial issues in soft tissue solitary fibrous tumors: a pathological and molecular review. Pathol Int.

[7] Kao YC, Lin PC, Yen SL (2016). Clinicopathological and genetic heterogeneity of the head and neck solitary fibrous tumours: a comparative histological, immunohistochemical and molecular study of 36 cases. Histopathology.

[8] Thompson LD, Lau SK (2018). Sinonasal tract solitary fibrous tumor: a clinicopathologic study of six cases with a comprehensive review of the literature. Head Neck Pathol.

[9] Demicco EG, Park MS, Araujo DM (2012). Solitary fibrous tumor: a clinicopathological study of 110 cases and proposed risk assessment model. Mod Pathol.

[10] Rodrigues RM, Fernandes AO, de Oliveira SP, Camisasca DR, Marques AA, Lourenço SQ (2017). Solitary fibrous tumor of the floor of the mouth. J Clin Exp Dent.

[11] Alkatan HM, Alsalamah AK, Almizel A (2020). Orbital solitary fibrous tumors: a multi-centered histopathological and immunohistochemical analysis with radiological description. Ann Saudi Med.

[12] Ganly I, Patel SG, Stambuk HE (2006). Solitary fibrous tumors of the head and neck: a clinicopathologic and radiologic review. Arch Otolaryngol Head Neck Surg.

[13] Park SJ, Lee YH, Lee KY, Oh KH, Kim Y (2016). A solitary fibrous tumor of the subglottic larynx: case report and literature review. Balkan Med J.

[14] Topaloglu O, Ucan B, Demirci T (2013). Solitary fibrous tumor of neck mimicking cold thyroid nodule in 99m tc thyroid scintigraphy. Case Rep Endocrinol.

[15] Tariq MU, Din NU, Abdul-Ghafar J, Park YK (2021). The many faces of solitary fibrous tumor; diversity of histological features, differential diagnosis and role of molecular studies and surrogate markers in avoiding misdiagnosis and predicting the behavior. Diagn Pathol.

[16] O'Regan EM, Vanguri V, Allen CM, Eversole LR, Wright JM, Woo SB (2009). Solitary fibrous tumor of the oral cavity: clinicopathologic and immunohistochemical study of 21 cases. Head Neck Pathol.

[17] Yuzawa S, Nishihara H, Wang L, Tsuda M, Kimura T, Tanino M, Tanaka S (2016). Analysis of NAB2-STAT6 gene fusion in 17 cases of meningeal solitary fibrous tumor/hemangiopericytoma: review of the literature. Am J Surg Pathol.

[18] Dagrada GP, Spagnuolo RD, Mauro V (2015). Solitary fibrous tumors: loss of chimeric protein expression and genomic instability mark dedifferentiation. Mod Pathol.

[19] Demicco EG, Wagner MJ, Maki RG, Gupta V, Iofin I, Lazar AJ, Wang WL (2017). Risk assessment in solitary fibrous tumors: validation and refinement of a risk stratification model. Mod Pathol.

[20] Thompson LD, Liou SS, Feldman KA (2021). Orbit solitary fibrous tumor: a proposed risk prediction model based on a case series and comprehensive literature review. Head Neck Pathol.

